# Development of a Predictive Algorithm for Suicidal Behavior Integrating Genetic Risk Markers and Digital Phenotyping: The Smartomics Study Protocol

**DOI:** 10.1192/j.eurpsy.2026.11263

**Published:** 2026-07-31

**Authors:** A. A. Porras-Segovia, C. Diaz-Tellez, L. Albarracín-García, E. Baca-García

**Affiliations:** 1 Health Research Institute Fundación Jiménez Díaz, Madrid; 2 Hospital Clinic, Barcelona , Spain

## Abstract

**Introduction:**

Each year, suicide claims approximately 700,000 lives worldwide and generates a significant financial burden. Integrating genomic data, exposomic factors, and digital phenotypes can enhance the development of short-term predictive models. Current knowledge and available tools provide the basis for designing personalized treatment strategies that incorporate real-time interventions to prevent suicide recurrence cost-effectively.

**Objectives:**

This study aims to develop a predictive algorithm for suicidal behavior integrating psychiatric assessments, genetic risk markers, digital phenotypes, and exposomic data.

**Methods:**

This protocol describes a retrospective multicenter study that will recruit participants with a clinical history of suicide across 25 hospitals across Spain with a catchment area of 8,6 million people (17,8% of Spain’s population). Our sample target is over 5,000 participants, ensuring 93.5% statistical power for genetic analysis. Eligible participants must be 18 years old and over or have parental consent if aged between 12 and 17. Data collection will include psychiatric assessments, biospecimen collections (DNA, RNA, plasma and serum), Google Takeout data for digital phenotyping, and a standardized set of administrative and clinical data registered for each patient. Genotyping will be performed with the Axiom Spanish array (>750,000 markers), and Genome-Wide Association Studies (GWAS) will be performed after genetic imputation in a whole sample of >10,000 individuals (5,000 suicide attempters; 5,000 controls). Prescription and clinical history will also be retrospectively integrated, and codified data statistics forms will periodically be sent to the Government. Statistical analyses will combine traditional regression models and AI-based algorithms to identify predictive behavioral, genomic profiles, and digital markers of suicidal behavior. Cost-effectiveness analyses of pharmacogenomic markers for antidepressant response will also be conducted.

**Results:**

By successfully implementing this project, we aim to reduce suicide rates, improve the quality of life for at-risk individuals, and lessen the emotional and economic burden on families and the healthcare system

**Conclusions:**

This study represents one of the most comprehensive efforts to date to integrate genomic, exposomic, and digital phenotyping data into suicide risk prediction. By combining large-scale genetic analyses with real-world clinical, behavioral, and environmental information, the project aims to generate a multidimensional predictive model capable of identifying individuals at imminent risk of suicidal behavior.

**Disclosure of Interest:**

None Declared

